# Differences in Energy Expenditures and Growth Dilution Explain Higher PCB Concentrations in Male Summer Flounder

**DOI:** 10.1371/journal.pone.0147223

**Published:** 2016-01-21

**Authors:** Charles P. Madenjian, Olaf P. Jensen, Richard R. Rediske, James P. O’Keefe, Anthony R. Vastano, Steven A. Pothoven

**Affiliations:** 1 U. S. Geological Survey, Great Lakes Science Center, Ann Arbor, Michigan, United States of America; 2 Department of Marine and Coastal Sciences, Rutgers University, New Brunswick, New Jersey, United States of America; 3 Annis Water Resources Institute, Grand Valley State University, Muskegon, Michigan, United States of America; 4 Michigan Department of Health and Human Services, Bureau of Laboratories, Lansing, Michigan, United States of America; 5 National Oceanic and Atmospheric Administration, Great Lakes Environmental Research Laboratory, Lake Michigan Field Station, Muskegon, Michigan, United States of America; Northwest Fisheries Science Center, NOAA Fisheries, UNITED STATES

## Abstract

Comparison of polychlorinated biphenyl (PCB) concentrations between the sexes of mature fish may reveal important behavioral and physiological differences between the sexes. We determined whole-fish PCB concentrations in 23 female summer flounder *Paralichthys dentatus* and 27 male summer flounder from New Jersey coastal waters. To investigate the potential for differences in diet or habitat utilization between the sexes, carbon and nitrogen stable isotope ratios were also determined. In 5 of the 23 female summer flounder, PCB concentrations in the somatic tissue and ovaries were determined. In addition, we used bioenergetics modeling to assess the contribution of the growth dilution effect to the observed difference in PCB concentrations between the sexes. Whole-fish PCB concentrations for females and males averaged 87 and 124 ng/g, respectively; thus males were 43% higher in PCB concentration compared with females. Carbon and nitrogen stable isotope ratios did not significantly differ between the sexes, suggesting that diet composition and habitat utilization did not vary between the sexes. Based on PCB determinations in the somatic tissue and ovaries, we predicted that PCB concentration of females would increase by 0.6%, on average, immediately after spawning due to release of eggs. Thus, the change in PCB concentration due to release of eggs did not explain the higher PCB concentrations observed in males. Bioenergetics modeling results indicated that the growth dilution effect could account for males being 19% higher in PCB concentration compared with females. Thus, the bulk of the observed difference in PCB concentrations between the sexes was not explained by growth dilution. We concluded that a higher rate of energy expenditure in males, stemming from greater activity and a greater resting metabolic rate, was most likely the primary driver for the observed difference in PCB concentrations between the sexes.

## Introduction

For seven species of fish, mature males have been shown to be 15–45% higher in whole-fish polychlorinated biphenyl (PCB) concentration than similarly aged mature females, and this difference has been primarily attributed to a higher rate of energy expenditure in mature males stemming from greater activity and a higher resting metabolic rate (or standard metabolic rate [SMR]) [1–3]. A higher rate of energy expenditure leads to a higher rate of food consumption, which, in turn, leads to a higher rate of PCB accumulation. These seven species included walleye *Sander vitreus*, lake trout *Salvelinus namaycush*, coho salmon *Oncorhynchus kisutch*, burbot *Lota lota*, sea lamprey *Petromyzon marinus*, cisco *Coregonus artedi*, and lake whitefish *Coregonus clupeaformis*, and therefore covered a wide gamut of feeding behaviors from planktivore to blood parasite to piscivore. All of the above-mentioned freshwater fish populations that have been surveyed for a difference in whole-fish PCB concentrations between the sexes were from the Laurentian Great Lakes region, except for the burbot population from Great Slave Lake (Northwest Territories, Canada). If females grow substantially faster than males, then a growth dilution effect may also contribute toward males having higher PCB concentrations than females, but this growth dilution effect has been estimated to be of relatively minor importance in most cases [1–2]. The contribution of the growth dilution effect to the observed difference in PCB concentrations between the sexes can be quantified using bioenergetics modeling.

Differences in PCB concentrations of muscle or liver tissue of fish between the sexes may not always accurately reflect the difference in whole-fish PCB concentrations between the sexes [1]. For example, in some fish species, females may exceed males in muscle PCB concentrations, whereas males exceed females in whole-fish PCB concentrations [1,3]. Thus, whole-fish PCB determinations are needed to be certain that observed differences in PCB concentrations between the sexes actually signal the above-mentioned differences in behavior and physiology between the sexes. Most studies addressing differences in PCB concentrations between the sexes of fish have been based on muscle tissue or liver tissue PCB determinations. Consequently, caution must be exercised in comparing studies based on muscle tissue or liver tissue PCB determinations with studies based on whole-fish PCB determinations. Whole-fish PCB determinations are useful in ecological studies [1], whereas muscle tissue PCB determinations are useful in setting fish consumption advisories [4].

In general, results from published studies based on muscle or liver tissue PCB determinations indicated that male fish exceeded female fish in PCB concentrations. For example, substantially greater PCB concentrations in the livers of male dab *Limanda limanda*, a species of flatfish, compared with female dab from the North Sea have been reported [5]. Similarly, significantly greater muscle PCB concentrations in males compared with females in both largemouth bass *Micropterus salmoides* and spotted bass *Micropterus punctulatus* from a southeastern USA reservoir have been documented [6]. Likewise, adult male European hake *Merluccius merluccius* had significantly higher PCB concentrations in both their muscle tissue and liver tissue compared with adult female European hake from the Mediterranean Ocean [7]. Moreover, muscle PCB concentrations were significantly greater in males than in females for most of the walleye populations surveyed in Canada [4]. However, in a number of cases, muscle tissue or liver tissue PCB concentrations did not significantly differ between the sexes; and, in a few cases, muscle tissue PCB concentration was significantly greater in females than in males. For example, significant differences in liver tissue PCB concentrations between the sexes of Atlantic cod *Gadus morhua* or European flounder *Platichthys flesus* caught in the Glomma River, a tributary to the North Sea, were not detected [8]. Similarly, muscle PCB concentrations from six species of freshwater fishes (other than walleye) in Canada rarely showed significant differences between the sexes [4]. Failure to detect a significant difference in PCB concentrations between the sexes, or finding significantly higher PCB concentrations in females compared with males, may simply have been artifacts, at least in part, of determining PCB concentrations in muscle tissue or liver tissue rather than in whole fish.

The summer flounder or fluke *Paralichthys dentatus* population supports the most important recreational and commercial flatfish fisheries of the U. S. Atlantic coast [9–10]. Geographic range of the stock and fishery extends from Massachusetts to North Carolina. Summer flounder have been highly sought by sport anglers in New York and New Jersey waters of the Atlantic Ocean [9–11]. Spawning season for summer flounder is protracted, extending from September to March, but peak spawning typically occurs during October and November. Diet of adult summer flounder consists primarily of fish and squid, and summer flounder is considered one of the important predators inhabiting the western Atlantic coastal ecosystem [12]. Female summer flounder grow substantially faster than male summer flounder [11]. To the best of our knowledge, differences in whole-fish PCB concentrations between the sexes of summer flounder have not been investigated. Documentation of males exceeding females in whole-fish PCB concentrations of summer flounder would represent an important milestone in assessing the pervasiveness of this apparently general characteristic of higher energy expenditure rate in males, because the summer flounder is a marine fish whereas the other fish populations that have been surveyed were from freshwater ecosystems.

The overall goal of our study was to evaluate the difference in whole-fish PCB concentrations between the sexes of summer flounder, and then identify the most likely explanations for the observed difference. Specific objectives included: (1) quantify the difference in whole-fish PCB concentrations between the sexes of summer flounder caught from a spawning aggregation off the New Jersey coast, (2) quantify the growth dilution effect on the difference in whole-fish PCB concentrations between the sexes using bioenergetics modeling, (3) quantify the difference between somatic tissue PCB concentration and ovary PCB concentration in female summer flounder, (4) estimate the change in whole-fish PCB concentration of female summer flounder associated with the release of eggs at spawning, and (5) evaluate potential differences in diet and habitat use between the sexes of summer flounder based on carbon and nitrogen stable isotope ratios between the sexes of summer flounder.

## Methods

### Field methods

Adult summer flounder were caught on 12 November 2013 by a commercial trawler fishing in New Jersey coastal waters about 30 km east of Barnegat Light, New Jersey. Bottom depth at the capture site was 18 m. We purchased 85 summer flounder from the commercial fisher (Viking Village) at Barnegat Light, New Jersey, and then transported the fish on ice in coolers to the Rutgers University Marine Field Station (RUMFS) in Tuckerton, New Jersey for further processing. Because the summer flounder were bought from a licensed commercial fisher, approval by an Institutional Animal Care and Use Committee was not needed. The commercial fishing boat was operated under a Shirred/Purse Seine, Otter/Beam Trawl License and a Summer Flounder Permit issued from the New Jersey Division of Fish and Wildlife (NJDFW) Bureau of Marine Fisheries (BMF), and Viking Village operated under a Summer Flounder Dealer Permit issued from the NJDFW BMF. At the RUMFS, total length of each summer flounder was measured to the nearest millimeter, and each summer flounder was weighed to the nearest gram. Sex and maturity of each summer flounder were determined via visual inspection of the gonads. Each summer flounder was then individually bagged with a cardboard tag marked with a unique identification number and then frozen at -20°C until further processing. This field study did not involve endangered or protected species.

### PCB, lipid, and stable isotope ratio determinations

Five female summer flounder were randomly selected for PCB determinations of both somatic tissue and ovaries. Each of these individuals was partially thawed, and otoliths were then removed for aging purposes. Ovaries were removed and weighed to the nearest g, and the remaining somatic tissue was also weighed to the nearest g. Ovaries and somatic tissue were homogenized separately in appropriately sized blenders. For the somatic tissue, about 100 g of the homogenate was placed in a contaminant-free glass jar, sealed with a lid, and then stored at -20°C. For the ovaries, all of the available homogenate (between 50 and 85 g) was placed in a contaminant-free glass jar, sealed with a lid, and then stored at -20°C. For the 18 remaining female summer flounder, each fish was partially thawed, and the otoliths were removed for aging purposes. Then, each whole fish was homogenized using appropriately sized blenders, and then approximately 100 g of the homogenate was placed in a contaminant-free glass jar, sealed with a lid, and stored at -20°C. We randomly selected 27 male summer flounder from the remaining 62 males, and we processed these 27 males in the same manner that we processed the 18 females used for whole-fish PCB determinations. The frozen homogenates were shipped to the Annis Water Resources Institute in Muskegon, MI for PCB determinations. The otoliths were transported to the National Oceanic and Atmospheric Administration (NOAA) Northeast Fisheries Science Center at Woods Hole, MA, where they were processed for age determination under the supervision of NOAA fishery scientists. Aging was accomplished via enumeration of annuli on thin-sectioned otoliths. For one of the summer flounder, otoliths were damaged during their removal, and therefore an age could not be assigned to this fish.

For each of 45 whole-fish homogenates and the 5 somatic tissue homogenates, about 5 g of the homogenate was transferred to a small plastic vial, then capped, and stored at -20°C until further processing. These samples were used to determine the carbon (δ^13^C) and nitrogen (δ^15^N) stable isotope ratios. Because the decision to determine stable isotope ratios in the homogenates was not made until after five summer flounder had already been through the entire homogenization process, stable isotope ratios were not determined for these five fish. Due to the limited amount of homogenate material for the ovaries, stable isotope ratios were not determined for the ovary tissues. Each homogenate was dried for approximately 12 h at 50°C in a drying oven, and then ground by hand with a mortar and pestle. Samples were analyzed for δ^13^C and δ^15^N at the University of California–Davis Stable Isotope Facility on a PDZ Europa ANCA GSL elemental analyzer (Elementar Analysensysteme GmbH, Hanau, Germany) in combination with a PDZ Europa 20–20 isotope ratio mass spectrometer (Sercon Ltd., Cheshire, United Kingdom). Delta values, in parts per thousand (‰), reported here are relative to V-PDB (Vienna Pee Dee Belemnite) for carbon and air for nitrogen.

At the Annis Water Resources Institute, we followed the procedures described by Carlson et al. [13] and Jude et al. [14] to determine concentrations of PCB congeners. In brief, homogenized fish tissue (20 g) and 40 g of sodium sulfate were placed in a pre-extracted fritted glass thimble after spiking with the surrogate standards (numbers 30, 61, 161, 127, and 166; AccuStandard, Inc., New Haven, CT, USA). The tissue was allowed to dry and then was extracted in a soxhlet for a minimum of 10 hours with 50:50 dichloromethane:hexane. Extracts were reduced to less than 2 mL using a Labconco Rapidvap (Labconco, Kansas City, MO, USA) and brought up to a 5 mL final volume with hexane. A 1-mL aliquot of the extract was used for gravimetric determination of lipids. A second 1-mL aliquot was passed through a column containing 5 g of 45% acidic silica gel (Kiesel gel, mesh size 230–400, Merck, Darmstadt, Germany) and a thin layer of sodium sulfate at the top. The column was cleaned with 10 mL of hexane prior to transferring the extracts. Samples then were eluted with 15 mL of hexane and concentrated as described above to a final volume of 1.0 mL.

Individual PCB congeners were identified and quantified with an Agilent 6890 gas chromatograph equipped with an electron capture detector (ECD) (Agilent Technologies, Santa Clara, CA, USA). A fused-silica DBXLB capillary column (60 m × 0.25 mm inside diameter, 0.25-μm film thickness) was used for separation. The oven temperature of the capillary column was programmed from 60 to 190°C at a rate of 20°C/min, then to 230°C at 1.5°C/min, then to 260°C at 5°C/min, and then to 300°C at 20°C/min. The final holding time was 8 min. Injector and detector temperatures were 300°C and 320°C, respectively. An external standard calibration method was used, with hydrogen as the carrier gas. Surrogate recoveries averaged 94 ± 0.9%. Procedural blanks were passed through the entire analytical procedure in order to determine if method related contamination or interferences were present. The gas chromatograph was calibrated with individual congener standards at five concentrations from AccuStandard (New Haven, CT, USA). Calibration accuracy was based on the ability to analyze aroclor standards and obtain predicted amounts and ratios obtained by Frame et al. [15]. The West Coast Fish Studies standard supplied by AccuStandard was used to perform additional calibration verification. Quality control samples (method blanks, matrix spikes, and duplicates) were analyzed to ensure precision and accuracy. Method blanks were run at a frequency of 1 per 20 samples and the mean (±SE) concentration was 0.47 (±0.07) μg/kg. Matrix spikes were also analyzed at a 5% frequency and mean recovery was 85 (±2.2)%. Matrix-spiked duplicates were analyzed at a 5% frequency and mean relative percent difference was 17 (±1.0)%. Detection limit for the individual PCB congeners was 0.025 ng/g, which was equal to five times the baseline noise. The lowest concentration for a calibration standard was 0.25 ng/g. All PCB determinations were completed within 11 months after capture of the fish.

We detected and quantified 77 single PCB congeners and 6 pairs of coeluting PCB congeners in the summer flounder homogenates (Table 1). Congener numbers were assigned according to Ballschmiter et al. [16]. Congener nomenclature was applied according to the International Union of Pure and Applied Chemistry (IUPAC) system [17]. For each homogenate, we summed all of the PCB congener concentrations to yield an estimate of total PCB concentration, ƩPCB. All PCB concentrations were expressed on a wet-weight basis. We also determined the lipid concentration of each homogenate using the procedure outlined by Schmidt and Hesselberg [18] and Hesselberg et al. [19]. Lipid concentrations were also expressed on a wet-weight basis. Results of all of the aforementioned determinations are provided in S1 File, S2 File and S3 File.

**Table 1:** List of PCB congeners that were detected and quantified in summer flounder caught in New Jersey coastal waters during November 2013.

Grouping	PCB congeners
**Dichloro:**	8
**Trichloro:**	18, 22, 25, 28, 31, 32, and 37
**Tetrachloro:**	41, 42, 44, 45, 46, 47, 48, 49, 52, 59, 60, 63, 64, 67, 70, 71, 74, and 75
**Pentachloro:**	84, 85, 87, 92, 95, 97, 99, 105, 110, 118, 119, 123, and 124
**Hexachloro:**	130, 131, 132, 134, 135, 138, 141, 146, 149, 151, 153, 156, 157, 158, 164, and 167
**Heptachloro:**	170, 171, 172, 173, 175, 176, 179, 180, 185, 187, 190, and 193
**Octachloro:**	194, 195, 196, 199, 200, 202, and 203
**Nonachloro:**	206 and 208
**Decachloro**:	209
**Trichloro coelutor pair:**	20/33
**Tetrachloro-pentachloro coelutor pairs:**	56/101 and 66/91
**Hexachloro-heptachloro coelutor pairs:**	128/174 and 163/178
**Heptachloro-octachloro coelutor pair:**	177/201

Congeners were numbered according to Ballschmiter et al. [16].

### Data analyses

To determine whole-fish ƩPCB of the five females selected for PCB determinations of ovaries and somatic tissue, the mass-balance approach used by Niimi [20] was followed. Following this approach, body burdens in the ovaries and somatic tissue were calculated and then summed, and this sum of the body burdens was then divided by the sum of the weights for the ovaries and somatic tissue to yield an estimate of whole-fish ƩPCB. This same approach was used to determine whole-fish concentrations of each of the PCB congeners as well as whole-fish lipid concentrations. In addition, using the procedure described by Niimi [20], the expected percent change in ƩPCB due to release of eggs at spawning was estimated for each of the five females by calculating the ratio of ƩPCB in the somatic tissue to the estimated whole-fish ƩPCB. The gonadosomatic index (GSI) was calculated for each of the five females by dividing the weight of the ovaries by the total weight of the fish and then multiplying by 100.

To determine whether whole-fish ƩPCB significantly differed between the sexes of summer flounder, we applied analysis of covariance (ANCOVA) to the summer flounder ƩPCB data. Five different ANCOVA models were initially considered, with whole-fish ƩPCB as the dependent variable, sex as the categorical variable, and age, total length (TL), weight, Fulton’s condition *K* (= weight ∙ 10^5^ ∙ TL^-3^), or lipid concentration of the summer flounder as the covariate. A sixth ANCOVA model was also considered, with whole-fish ƩPCB as the dependent variable, sex as the categorical variable, and age, TL, weight, *K*, and lipid concentration as the five covariates. All covariates were found to be insignificant (*P* > 0.10) in the six ANCOVA model applications, except for lipid concentration. Thus, the ANCOVA model with lipid concentration as the covariate was used to test whether sex had a significant effect on whole-fish ƩPCB. To quantify the difference in whole-fish ƩPCBs between the sexes, we substituted the mean values of lipid concentration for both sexes into the fitted ANCOVA model to generate estimates of mean ƩPCB for both sexes, and then we calculated the ratio of the ƩPCB for the sex with the higher ƩPCB to the ƩPCB for the sex with the lower ƩPCB. The carbon and nitrogen stable isotope ratios were not considered as potential covariates in ANCOVA models, because stable isotope data were missing for five fish and we opted to use the maximum amount of ƩPCB data possible to characterize the difference in ƩPCBs between the sexes. To determine whether lipid concentration varied significantly between the sexes of summer flounder, a two-sample *t* test was applied to whole-fish lipid concentration data. We set α = 0.05 for all of our statistical testing. All of the statistical analyses were performed using SAS 9.4 (SAS Institute, Cary, NC, USA).

To determine whether PCB congener distributions varied between the sexes of summer flounder, we first determined the PCB congener distribution for each of the 50 fish by calculating the proportion of whole-fish ƩPCB represented by each of the single PCB congeners and coelutor pairs. Next, the PCB congeners were grouped by homologs, and the proportion of ƩPCB represented by each of these groups was calculated for each of the 50 fish. Because only one dichloro congener was detected in the summer flounder (Table 1), the dichloro and trichloro congeners were lumped into a single group, which also included the 20/33 coelutor pair. Similarly, the nonachloro congeners and decachloro congener were lumped into a single group. The 56/101 and 66/91 coelutor pairs were placed into a tetrachloro-pentachloro group, the 128/174 and 163/178 coelutor pairs were placed into a hexachloro-heptachloro group, and the 177/201 coelutor pair was placed into a heptachloro-octachloro group. These coelutor pair groups were separate from the tetrachloro, pentachloro, hexachloro, heptachloro, and octachloro groups. Thus, the total number of groups for principal component analysis was 10. Principal component analysis was applied to the proportions of whole-fish ƩPCB in these 10 groups, and the plot principal component 2 versus principal component 1 was visually inspected to determine whether the sexes could be clearly separated. In addition, we performed a battery of two-sample *t* tests to determine whether the proportion of ƩPCB represented by each of the single PCB congeners and coelutor pairs varied significantly between the sexes. A Bonferroni correction was applied to the α level for this battery of *t* tests such that a difference was considered significant when *P* < 0.05/83 or 0.0006. Comparison of PCB congener distributions between the sexes may be useful in inferring differences in habitat utilization or diet composition between the sexes [21–22].

To determine whether δ^13^C and δ^15^N differed significantly between the sexes of summer flounder, two-sample *t* tests were applied to the carbon and nitrogen stable isotope data. In aquatic ecosystems, δ^13^C can be used to help differentiate between the two major sources of available energy, namely the littoral (nearshore) production from benthic algae and detritus and the pelagic (open water) production from phytoplankton [23–24]. The base of the littoral food web tends to be enriched in ^13^C (less negative δ^13^C) compared with the base of the pelagic food web. The stable isotope ratio for nitrogen, δ^15^N, can be used to estimate trophic position because the δ^15^N for a consumer is typically enriched by 3–4‰ relative to its diet [23–24]. Thus, δ^13^C and δ^15^N determinations may provide some insight into whether the sexes differed in habitat utilization and diet composition.

### Bioenergetics modeling

Bioenergetics modeling was used to quantify the growth dilution effect on the difference in ƩPCBs between the sexes of summer flounder, following the approach proposed by Madenjian [1]. To briefly review, gross growth efficiency (GGE) is equal to growth divided by the amount of food needed to attain that growth, and ƩPCB is inversely proportional to GGE. Thus, if the difference in ƩPCBs between the sexes is solely due to a difference in GGE between the sexes, then the ratio of ƩPCB of males to ƩPCB of females should be equal to the ratio of GGE for females to GGE for males. For example, if males were 20% greater in ƩPCB than females, and the difference in ƩPCBs between the sexes was solely attributable to a difference in GGEs between the sexes, then females should be 20% greater in GGE than males. Bioenergetics modeling is used to quantify the relative difference in GGEs between the sexes that is solely due to a difference in growth rates between the sexes, and this quantification represents the magnitude of the growth dilution effect [1]. In performing this bioenergetics modeling, SMR and activity do not vary between the sexes, because the objective is to quantify the relative difference in GGEs between the sexes that is solely due to a difference in growth rates between the sexes [1].

To develop growth trajectories for both female and male summer flounder, a von Bertalanffy growth model was fitted to the age and TL data for each sex. In addition, a length-weight regression was fitted to TL and weight data for each sex. Fitted von Bertalanffy growth models and length-weight regressions were provided by NOAA fishery scientists from the Northeast Fisheries Science Center (NEFSC; Mark Terceiro *NOAA NEFSC*, personal communication). These data were based on results from the NEFSC bottom trawl survey conducted in the fall during years 2005–2014. Based on the fitted von Bertalanffy growth curves, TL at age was estimated for females and males over ages 0 through 8. The oldest summer flounder from our sample 50 fish was an age-8 individual. The fitted length-weight regressions were then used to estimate weight at age over the same age range. Because 1 October was the typical midpoint of the time schedule for deployment of the NEFSC fall bottom trawl survey, we designated 1 October as the starting day in our bioenergetics model simulations. Estimation of food consumption by an age-0 fish, via bioenergetics modeling, from time of hatching to 1 October was not feasible due to insufficient diet composition data. Thus, we assumed that GGE over this time period was equal to 0.20, which was the estimate of GGE for age-0 summer flounder from laboratory experiments [25], for both females and males.

We used the bioenergetics model developed by Burke and Rice [26] for southern flounder *Paralichthys lethostigma*, a species closely related to the summer flounder, to estimate food consumption by an average female summer flounder and an average male summer flounder. This bioenergetics model represented an energy budget for the fish. Energy losses were calculated as a function of water temperature, summer flounder size (weight), and consumption rate, and then an estimate of the amount of food consumption necessary for a summer flounder to achieve the observed size (weight) at a given age was generated, using the bioenergetics model. The energy budget of the summer flounder can be written as: *C* = *G* + *R* + *Eg* + *Ex* + *S*, where *C* = consumption, *G* = growth, *R* = respiration, *Eg* = egestion, *Ex* = excretion, and *S* = spawning losses. Respiration was modeled as a function of water temperature and summer flounder weight. Egestion and excretion were modeled as functions of food consumption. Spawning was simulated by the summer flounder losing the appropriate amount of weight on the day of spawning. Mature males lost 1.7% of their body weight on the spawning day, based on GSI measurements for male summer flounder in ripe condition [27] (David McElroy *NOAA NEFSC*, personal communication). For mature females, we simulated a loss of body weight equivalent to the mean GSI for the five females from our study. We designated 15 November as the spawning day in our bioenergetics model simulations, based on advice from NOAA fishery scientists (Mark Terceiro, personal communication). Summer flounder were assumed to reach maturity at age 1 in the fall [28]. The bioenergetics model was coded into a Turbo Pascal 6.0 (Borland International, Scotts Valley, CA, USA) computer program, and the computer program was then run to generate estimates of food consumption.

Additional bioenergetics model inputs included the water temperature regime experienced by summer flounder, diet composition for summer flounder, and energy densities of both prey and the summer flounder. The water temperature regime was taken from Narváez et al. [29]. Diet composition for summer flounder was taken from Wuenschel et al. [12]. The 11 prey categories included anchovies *Anchoa* spp., sand lances *Ammodytes* spp., weakfish *Cynoscion regalis*, round herring *Etrumeus teres*, American butterfish *Peprilus triacanthus*, crabs, mysids and shrimp, other crustaceans, squid (primarily *Loligo* spp.), other molluscs, and miscellaneous (nematodes, polychaetes, annelids, leeches, eggs, and insects). Prey energy densities were taken from Steimle and Terranova [30], Hartman and Brandt [31], and Rudstam [32]. To estimate the energy density of summer flounder, five homogenates of males and five homogenates of females were randomly selected for energy density determinations. For each homogenate, energy density was determined using a Parr 1261 isoperibol bomb calorimeter (Parr Instrument Company, Moline, IL, USA), following the procedure outlined by Pothoven et al. [33]. Energy densities were expressed on a wet-weight basis. Energy densities did not vary between the sexes and showed no trend with increasing summer flounder weight. Thus, the energy density of mature summer flounder was assumed to be equal to 5795 J/g, the average value for the 10 energy density determinations. In contrast, from an earlier study, energy density of immature summer flounder was estimated to be 4291 J/g [34]. Using the fitted von Bertalanffy growth models and the fitted length-weight regressions, weight at first maturity was estimated to be 379 g. We assumed that summer flounder energy density linearly increased from 4291 to 5795 J/g as summer flounder weight increased from 0 to 379 g. The slope of this line was 3.968 J g^-1^ g^-1^. Again, energy density was assumed to be 5795 J/g for summer flounder ≥ 379 g in weight.

Using the food consumption estimates from the bioenergetics modeling, cumulative GGE for each sex was calculated by dividing the increase in summer flounder weight by the cumulative amount of food consumption required to attain the observed growth. The ratio of cumulative GGE for females to cumulative GGE for males was then calculated for each of the summer flounder ages 0–8. As previously mentioned, if the difference in ƩPCBs between the sexes were to be fully explained by the growth dilution effect, then the ratio of GGE for females to GGE for males would equal the ratio of ƩPCB of males to ƩPCB of females.

## Results

### Difference in whole-fish ƩPCBs between the sexes

Despite the age distributions strongly overlapping between the sexes, female summer flounder were substantially larger than male summer flounder (Table 2). Ages for females ranged from 2 to 6 years, and ages for males ranged from 3 to 8 years. Mean age for males (4.2 years) was slightly higher than mean age for females (3.7 years). Females averaged 497 mm in TL, whereas males averaged 433 mm in TL. Females averaged 1264 g, whereas males averaged 863 g (Table 2). All summer flounder gonads were ripe or nearly ripe.

**Table 2:** Mean values for total length, weight, age, lipid concentration, δ^13^C, and δ^15^N, by sex, of the summer flounder from New Jersey coastal waters, November 2013, used in the study.

Characteristic	Females, n	Females, Mean	Males, n	Males, Mean
**Total length (mm)**	23	497 (7)	27	433 (7)
**Weight (g)**	23	1264 (53)	27	863 (48)
**Age (years)**	23	3.7 (0.2)	26	4.2 (0.2)
**Lipid concentration (%)**	23	3.5 (0.3)	27	3.7 (0.2)
**δ**^**13**^**C (‰)**	22	-18.1 (0.3)	23	-18.4 (0.2)
**δ**^**15**^**N (‰)**	22	14.9 (0.2)	23	14.6 (0.1)

Standard error of the mean enclosed within parentheses. n = number of fish.

**Fig 1:**
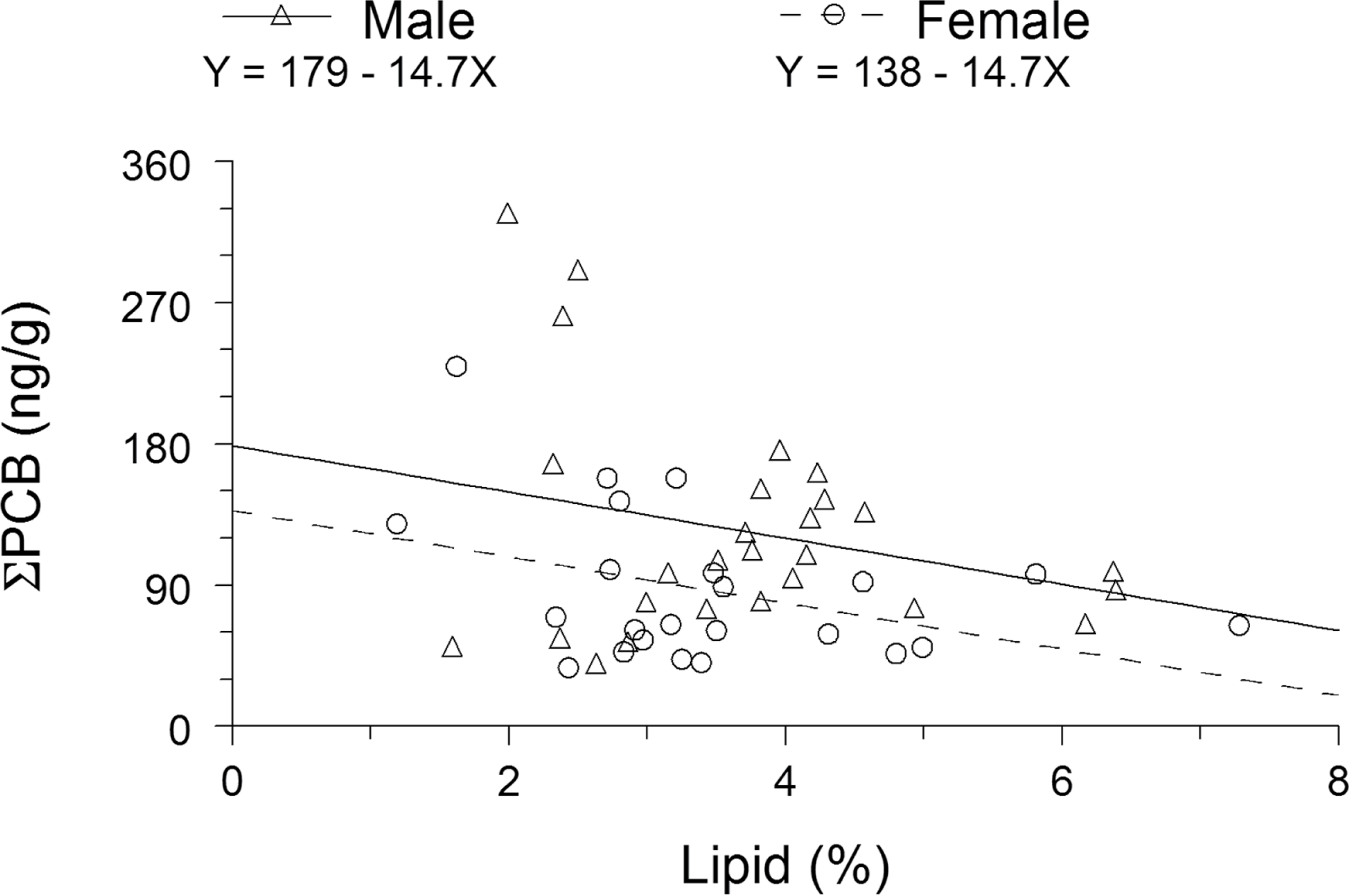
Whole-fish ƩPCB as a function of lipid concentration for summer flounder from New Jersey coastal waters, November 2013. Displayed lines are from the fitted ANCOVA model.

Males were significantly higher in whole-fish ƩPCB than females (ANCOVA: *F* = 5.82; df = 1,47; *P* = 0.0198), and whole-fish ƩPCB significantly decreased with increasing lipid concentration (ANCOVA: *F* = 4.89; df = 1,47; *P* = 0.0319) (Fig 1). Substituting mean lipid concentrations for females and males into the fitted ANCOVA model yielded estimates of mean ƩPCB of 86.8 and 124.3 ng/g for females and males, respectively. Thus, on average, males were 43.3% higher in ƩPCB than females. The assumption of homogeneity of slopes for the ANCOVA application was met (*F* = 0.05; df = 1,46; *P* = 0.8243). Lipid concentration did not significantly differ between the sexes (*t* test: *t* = -0.67; df = 48; *P* = 0.5220).

### ƩPCB in ovaries and somatic tissue

Mean ƩPCB in somatic tissue slightly exceeded mean ƩPCB in ovaries (Table 3). On average, whole-fish ƩPCB of the females was expected to increase 0.6% immediately after spawning due to release of eggs. Mean GSI for the females was 4.3% (Table 3). Lipid concentration in the ovaries was slightly higher than that in somatic tissue.

**Table 3:** Mean values for ƩPCB in ovaries, ƩPCB in somatic tissue, expected percent change in whole-fish ƩPCB immediately after spawning due to release of eggs, gonadosomatic index (GSI), lipid concentration in ovaries, and lipid concentration in somatic tissue of the five female summer flounder from New Jersey coastal waters, November 2013, used in the study.

Characteristic	Mean
**ƩPCB in ovaries (ng/g)**	71.9 (23.0)
**ƩPCB in somatic tissue (ng/g)**	83.7 (20.9)
**Expected percent change in whole-fish ƩPCBimmediately after spawning due to release of eggs (%)**	+0.6 (0.4)
**GSI (%)**	4.3 (0.4)
**Lipid concentration in ovaries (%)**	5.0 (0.3)
**Lipid concentration in somatic tissue (%)**	4.0 (0.5)

Standard error of mean enclosed within parentheses.

### Difference in PCB congener distributions between the sexes

**Fig 2:**
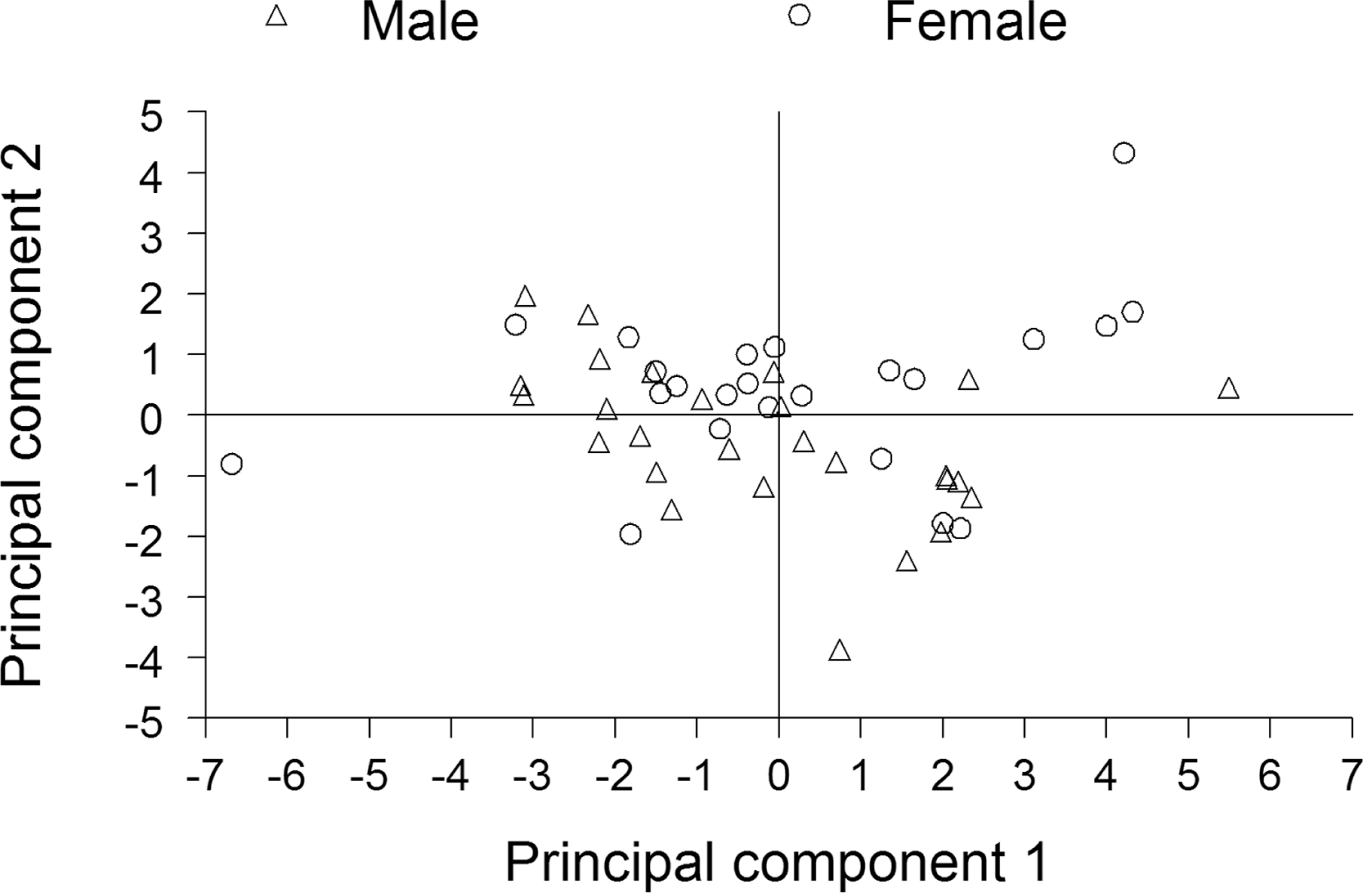
Principal component 2 versus principal component 1, based on application of principal component analysis to proportions of ƩPCB in 10 homolog groups. The PCB congener determinations were for summer flounder from New Jersey coastal waters, November 2013.

Based on results from principal component analysis, PCB congener distributions could not be separated by sex (Fig 2). Similarly, results from the battery of *t* tests indicated very little difference in PCB congener distributions between the sexes. Of the 83 *t* tests, only 3 yielded a significant difference. The proportions of ƩPCB represented by PCB congeners 44 and 132 were significantly higher in females compared with males, whereas the proportion of ƩPCB represented by PCB congener 48 was significantly higher in males compared with females. These 3 congeners were minor contributors to ƩPCB, as PCB congeners 44 and 48 each represented less than 0.2% of ƩPCB, and PCB congener 132 represented less than 1% of ƩPCB.

### Difference in δ^13^C and δ^15^N between the sexes

Stable isotope ratios did not significantly differ between the sexes for both carbon (*t* test: *t* = 0.76; df = 43; *P* = 0.4520) and nitrogen (*t* test: *t* = 1.59; df = 43; *P* = 0.1192). Mean δ^13^C values for females and males were -18.1 and -18.4‰, respectively (Table 2), while mean δ^15^N values for females and males were 14.9 and 14.6‰, respectively.

### Bioenergetics modeling

Females grew faster than males. Based on the fitted von Bertalanffy growth models and length-weight regressions, mean weights at ages 2, 4, 6, and 8 were 702, 1627, 2641, and 3570 g, respectively, for females. In contrast, mean weights at ages 2, 4, 6, and 8 were 543, 1015, 1457, and 1819 g, respectively. Thus, at age 8, size (weight) of females was nearly double that of males.

**Fig 3:**
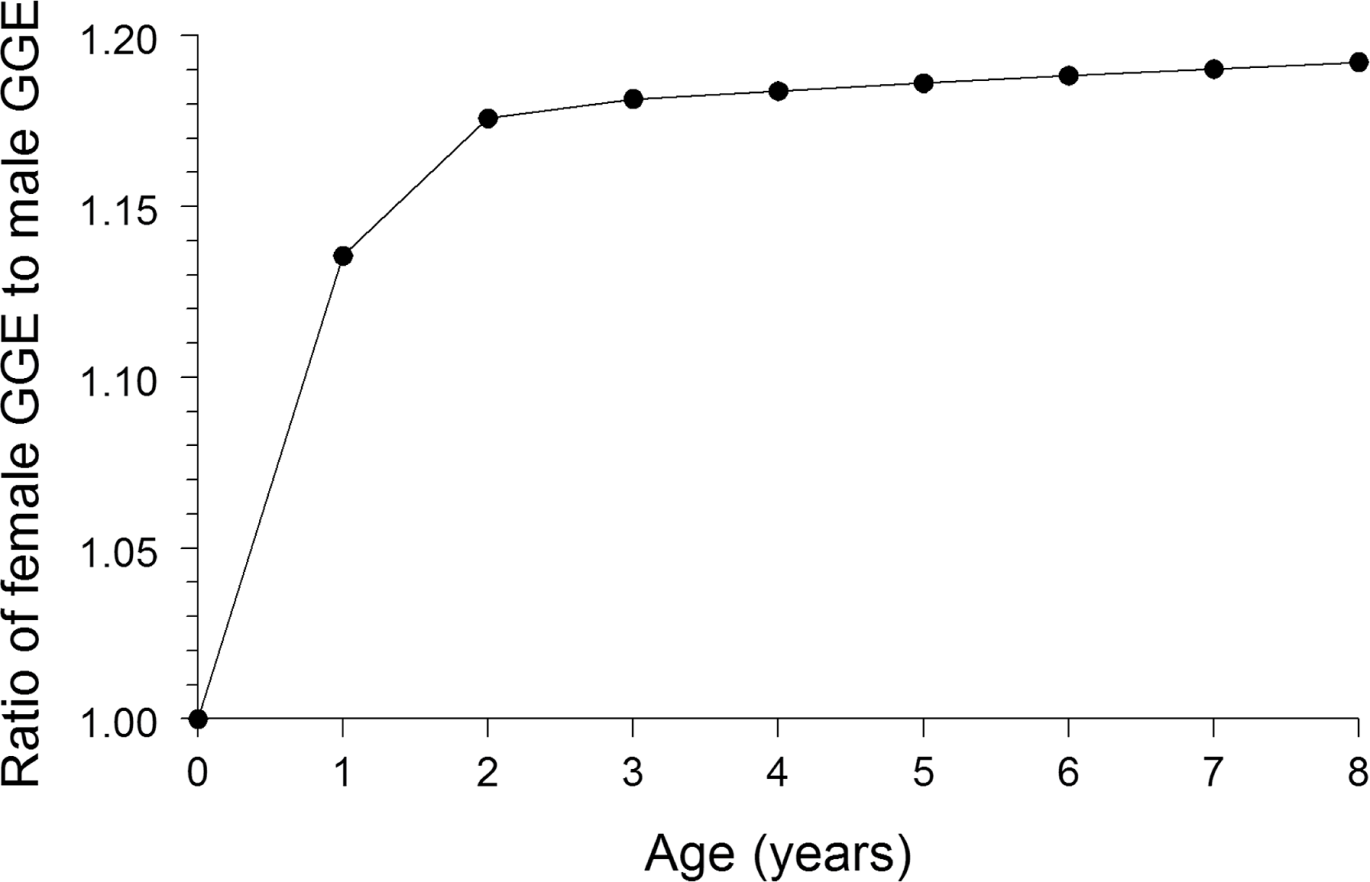
Estimated ratio of cumulative gross growth efficiency (GGE) of females to cumulative GGE of males, as a function of age, for summer flounder from New Jersey coastal waters, November 2013. Estimates based on application of the southern flounder bioenergetics model by Burke and Rice [26].

The ratio of GGE for females to GGE for males monotonically increased as age increased from 0 to 8 years (Fig 3). Across ages 2–8 years, the ratio of GGE for females to GGE for males averaged 1.185. Thus, the growth dilution effect could account for males being 18.5% greater in ƩPCB than females.

## Discussion

Change in ƩPCB due to release of eggs at spawning had no explanatory power whatsoever with regard to the 43% higher ƩPCB observed in male summer flounder compared with female summer flounder. We estimated that females would increase in ƩPCB by 0.6%, on average, immediately after spawning due to release of eggs, whereas males were significantly higher in ƩPCB compared with females. Because ƩPCB in females was expected to increase immediately after spawning, the release of eggs by females could not, in any way, explain the higher ƩPCB observed in males compared with females. Madenjian [1] contended that, in most fish populations, the change in ƩPCB of females immediately after spawning due to release of eggs was not a factor contributing to the higher ƩPCB observed in males compared with females, and our results supported that contention. Summer flounder shared this characteristic of females slightly increasing in ƩPCB immediately after spawning due to shedding of eggs with walleye, lake trout, rainbow trout *Oncorhynchus mykiss*, white sucker *Catostomus commersoni*, and lake whitefish [1,3]. In addition, PCB determinations for coho salmon and sea lamprey provided further support for the contention that release of eggs was not responsible for the greater PCB concentrations observed in males compared with females [35–36]. In both of these studies, the coho salmon and sea lamprey populations were sampled prior to spawning. Both species are semelparous in that they spawn just once during their lifetime and then die soon thereafter. Thus, these fish had never spawned prior to being caught, and therefore release of eggs at spawning could not possibly explain the greater ƩPCB observed in males compared with females for both species.

Although results from the bioenergetics modeling indicated that the growth dilution effect could account for males being 18.5% greater in ƩPCB compared with females, we found that male summer flounder were 43.3% greater in ƩPCB than female summer flounder. Thus, even though a substantial portion (18.5/43.3 or 43%) of this difference in ƩPCBs between the sexes of summer flounder was attributable to the growth dilution effect, most of the observed difference in ƩPCBs between the sexes was unexplained by the growth dilution effect. In contrast, the growth dilution effect accounted for less than 3% of the observed difference in ƩPCBs between the sexes of lake trout, coho salmon, burbot, sea lamprey, and lake whitefish [3,35–38]. For walleye, the growth dilution effect could explain males being about 10% greater in ƩPCB than females, and therefore the growth dilution effect accounted for 10/34 or 29% of the observed difference in ƩPCBs between the sexes [39]. For cisco, the growth dilution effect accounted for 3/43 or 7% of the observed difference in ƩPCBs between the sexes [2]. Thus, in most cases, the growth dilution effect was a very minor contributor to the observed difference in ƩPCBs between the sexes.

A “hot spot” effect, as described by Madenjian [1], did not appear to contribute to the observed difference in ƩPCBs between the sexes of summer flounder, for three reasons. A “hot spot” effect is realized when: (1) a PCB “hot spot”, where sediment PCB concentrations are sufficiently elevated to cause an elevation in the PCB concentrations of the prey, is located within the range occupied by the fish population, and (2) fish of one sex are spatially segregated from fish of the other sex for part of year such that fish of one sex spatially overlap with prey of relatively high PCB concentration associated with the PCB “hot spot”, whereas fish of the other sex spatially overlap with prey of relatively low PCB concentration. Both of these conditions must be met for a “hot spot” effect to occur. The first reason for contending that evidence to support a “hot spot” effect was lacking was that the PCB congener distributions did not appreciably vary between the sexes of summer flounder. A “hot spot” effect has typically been associated with a substantial difference in PCB congener distributions between the sexes [21]. In contrast, we found a minimal amount of variation in the PCB congener distributions between the sexes of summer flounder. The second reason was that both δ^13^C and δ^15^N did not significantly vary with sex. These stable isotope ratio results suggested that both habitat utilization and diet composition did not appreciably vary between the sexes of summer flounder. The third reason was that the relative difference in ƩPCBs between the sexes of summer flounder was not indicative of a “hot spot” effect. In previous studies, a “hot spot” effect has been associated with males being 1.7 to 2.5 times higher ƩPCB than females [1,21]. However, we determined that male summer flounder were 1.43 times higher in ƩPCB than female summer flounder. Further, over 40% of this difference in ƩPCBs between the sexes was attributable to the growth dilution effect. Thus, the relative difference in ƩPCBs between the sexes of summer flounder fell within the range of relative differences in ƩPCBs reported for the seven aforementioned species of freshwater fishes. These results suggested that a “hot spot” effect was an unlikely contributor to the observed difference in ƩPCBs between the sexes of summer flounder, because the “hot spot” effect was not identified as a likely contributor to the observed differences in ƩPCBs between the sexes of these seven freshwater fishes [1–3].

Despite the paucity of evidence for a “hot spot” effect on the summer flounder ƩPCB, a few PCB “hot spots” still persist in the sediments of certain estuarine areas, including the New York–New Jersey Harbor estuary and the Delaware River estuary [40–41]. In addition, the Historic Area Remediation Site (HARS), located about 10 km east of the base of Sandy Hook (New Jersey), continues to be remediated [42], although the remediation is more than halfway completed (S. Knowles *U*. *S*. *Army Corps of Engineers*, personal communication). Dredged material from the New York–New Jersey Harbor, garbage, city refuse, cellar dirt (natural rock and soil excavated during building construction), and sediments had been dumped offshore of Sandy Hook during the late 1800s through 1997. In 1997, the U. S. Environmental Protection Agency (USEPA) closed this area offshore of Sandy Hook to any further dumping, and designated it the HARS. Beginning in 1998, a plan was implemented to reduce environmental impacts at this site to acceptable levels by capping the sediments with at least 1 m of uncontaminated dredged material [42]. By 2014, about 65% of the designated remediation area had been capped (S. Knowles, personal communication). The HARS was located about 70 km north of the capture area for the summer flounder used in our study.

We concluded that the primary driver of the 43% higher ƩPCB in male summer flounder compared with female summer flounder was most likely a higher rate of energy expenditure by males, stemming from greater activity and a greater SMR. This higher rate of energy expenditure led to a higher rate of food consumption, which in turn led to a higher PCB accumulation rate. In the few cases where differences in activities between the sexes of fish have been documented, males have been shown to have greater activity compared with females [2,36]. Similarly, higher SMR in males compared with females has been documented for brown trout *Salmo trutta*, *Sebastolobus altivelis*, and largemouth bass *Micropterus salmoides* [43–45]. Further, Lozán [46] reported a 15% higher SMR in male dab compared with female dab. A greater rate of energy expenditure by males has been hypothesized as a characteristic common to most fish populations around the world [1].

Our findings have implications for both basic and applied sciences. We have shown that males have significantly higher whole-fish ƩPCB than females in a population of summer flounder, a marine fish, thereby corroborating and extending the results from earlier studies on freshwater fishes, including walleye, lake trout, coho salmon, burbot, sea lamprey, cisco, and lake whitefish. The emergence of this pattern of higher whole-fish ƩPCB in males continues, as this pattern appears to hold for both freshwater and saltwater fishes. We envision that this new line of research will eventually be useful in developing sex-specific bioenergetics models for fish. From an applied science perspective, this new research on differences in whole-fish ƩPCBs between the sexes of fish could not only be used to develop more efficient strategies for monitoring ƩPCB in fish populations [47], but may also prove useful in developing fish consumption advisories [4].

Although lipid concentration was once believed to be the primary regulator of PCB accumulation in fish, food consumption is now recognized as the governing factor [48–50]. In general, lipid concentration is positively correlated with ƩPCB, although the relationship is not a cause-and-effect one [49]. Thus, the observed difference in ƩPCBs between the sexes of summer flounder was not expected to be due to a difference in lipid concentration between the sexes. Males were slightly higher in lipid concentration than females, but the difference was not significant. In burbot from Great Slave Lake, females were higher in lipid concentration, but males were higher in ƩPCB [38]. Interestingly, we found that summer flounder ƩPCB actually decreased with increasing lipid concentration. Reasons for this decrease were unclear. Perhaps prey with relatively high lipid concentration were also relatively low in ƩPCB.

Our conclusion that males expended energy at a higher rate than females, and therefore consumed food at a higher rate, was partially dependent on the assumption that the bulk of PCBs accumulated by the summer flounder entered the bodies of the fish via dietary intake rather than direct uptake from the water. Based on results from previous studies, this assumption appeared to be sound. Less than 1% of the PCB body burden in adult lake trout from Lake Michigan entered the lake trout via direct uptake from the water whereas the rest of the PCB body burden was from food consumption, according to the results of a PCB accumulation model applied to field data [50]. Further, the field estimate of net trophic transfer efficiency of PCBs to Lake Michigan lake trout from their prey was within 1% of the laboratory estimate of net trophic transfer efficiency of PCBs to lake trout from their food [51]. The field estimate of net trophic transfer efficiency was based on the assumption that direct uptake of PCBs from water was negligible. Thus, the fact that the field estimate was nearly identical to the laboratory estimate indicated that direct uptake of PCBs from water was practically negligible. Similarly, the field estimate of net trophic transfer efficiency of PCBs to Lake Michigan lake whitefish from their prey was within 6% of the laboratory estimate of net trophic transfer efficiency of PCBs to lake whitefish from food, suggesting that direct uptake of PCBs from water was of very minor importance in the PCB budget for lake whitefish [52]. In addition, direct uptake of PCBs from water may be even less important for summer flounder in the Atlantic Ocean than for lake trout or lake whitefish in Lake Michigan. Mean PCB concentration in Lake Michigan water during the 1990s was estimated to be 0.64 ng/L [53], whereas PCB concentrations in water from the North Atlantic during the 1990s averaged 0.0003 ng/L [54]. Because the rate of direct uptake from water is directly proportional to PCB concentration in the water [50], the rate of direct uptake of PCBs from the water for a fish inhabiting the North Atlantic may be substantially less than that for a fish in Lake Michigan. Although presumed to be a benthic species, the summer flounder may actually spend a considerable amount of time in the water column [12]. Finally, Carlson and Hites [55] concluded that the bulk of the PCBs accumulated by farmed Atlantic salmon *Salmo salar* was from dietary intake, because the PCB congener distribution in the Atlantic salmon closely corresponded to the PCB congener distribution in the commercial feed fed to the Atlantic salmon.

Conclusions drawn from our bioenergetics modeling results were robust to uncertainties in the model inputs. The growth dilution effect was defined as the relative difference in GGEs between the sexes solely due to a difference in growth rates between the sexes. We would expect that the degree of bias in GGE estimates arising from inaccuracies in the values used for model inputs should be similar for both sexes. Thus, the ratio of GGE in females to GGE in males should be relatively unaffected by these biases. In addition, Burke and Rice [26] reported that their bioenergetics model predictions were relatively insensitive to perturbations in the values of most of the model parameters. Further, the remaining model parameters, for which perturbations in their values had a relatively large effect on bioenergetics model predictions, were often associated with a high degree of confidence in their estimated values. Finally, Burke and Rice [26] concluded that their bioenergetics model estimates of food consumption by southern flounder held in a pond during a feeding experiment were reasonably accurate, as the model estimates of cumulative food consumption were within 5% of observed cumulative food consumption.

A considerable amount of additional research will be needed before a credible attempt to model PCB accumulation in fish of both sexes of summer flounder from New Jersey coastal waters can be made. The issue of whether females show some degree of spatial separation from males over extended periods of time still needs to be fully resolved. Data from the commercial and recreational fishery catches during 2009–2011 provided evidence that summer flounder exhibit some degree of sex-specific segregation [11,56]. Based on results of their data analyses, Morson et al. [11,56] proposed that females are more likely to spend their summers relatively close to shore compared with males. However, even if this inferred spatial separation were verified by years of additional fieldwork, it still may not explain the higher ƩPCB in males. During the early fall, and presumably during the summer as well, the summer flounder population can range more than 50 km east of the New Jersey shoreline [9]. Thus, this proposed “close to shore” area could potentially extend well beyond 10 km from the shoreline. If so, then this area would not only encompass the aforementioned PCB “hot spots” in estuaries but would also include the HARS. Results from our carbon stable isotope determinations did not indicate spatial separation of females from males, however such determinations may not provide sufficient resolution to this issue. Carbon stable isotope analysis has the potential to distinguish between the primary sources of carbon in the food chain of two or more groups of organisms, however this technique has limitations in accurately identifying the primary source of carbon in the food chain [23,57]. If some degree of spatial separation of female summer flounder from male summer flounder were verified by additional fieldwork, the question of whether fish of one sex spend more time in areas with elevated sediment PCB concentrations than fish of the other sex would still need to be answered. Again, although the stable isotope and PCB congener distribution results do not support this possibility, the issue still needs to be fully resolved. Certainly, it is possible that diet composition may be identical among two groups of spatially separated fish, whereas ƩPCB of the prey may vary substantially between the two groups. To accurately model PCB accumulation in fish of both sexes of summer flounder, PCB concentration of the water specifically inhabited by the summer flounder would have to be determined. Summer flounder apparently do move about the water column [12], but some time is also spent on ocean bottom. Thus, PCB concentration of the water column inhabited by the summer flounder, as well as PCB concentration of the water surrounding the summer flounder when the fish are on ocean bottom, must be known to accurately assess direct uptake of PCBs from water. Moreover, amounts of time spent on bottom and in the water column would also have to be taken into account. In areas where sediment PCB concentration is elevated, PCB concentrations in the pore water of the sediments and in water overlying the sediments can also be relatively high [58–60]. The importance of direct uptake of PCBs from water in the PCB budget for a fish depends on the ratio of PCB concentration in food to PCB concentration in water [61]. If PCB concentration in water is sufficiently high relative to PCB concentration of food, direct uptake of PCBs from water would be an important component of the PCB budget. Given that direct uptake of PCBs from water is positively influenced by the ventilation rate of the fish [61], the rate of direct uptake of PCBs from water would be expected to be higher in males than in females, because males are believed to expend energy at a faster rate than females. Finally, whole-organism PCB concentration of the various prey items would also have to be determined to accurately model PCB accumulation in summer flounder.

One new research direction suggested by our study would be to more accurately assess the difference in energy expenditure rates between the sexes of summer flounder. Both active and passive telemetry, using acoustic tags attached to fish, has provided the means to track movements of summer flounder into and out of estuarine areas, as well as within estuarine areas [62–64]. However, to date, differences between the sexes have not been addressed. Telemetry using acoustic tags may afford researchers the opportunity to quantify the difference in activities between the sexes of summer flounder, but, of course, researchers would have to be able to sex the fish to successfully complete these types of investigations. To accurately quantify activity, estimates of swimming speed and/or total distance traveled over time are needed, and therefore movements of the summer flounder would have to be tracked at a fine geographic scale. Use of accelerometer tags may provide another means of assessing the difference in activities between the sexes of summer flounder [65–66]. In addition, laboratory respirometry could be used to quantify the difference in SMRs between the sexes of summer flounder.

## Conclusions

We determined that, on average, whole-fish ƩPCB in adult male summer flounder exceeded whole-fish ƩPCB in adult female summer flounder by 43%. We concluded that this difference was most likely due to a combination of higher energy expenditure rate in males, stemming from greater activity and a higher SMR, and the growth dilution effect. This higher rate of energy expenditure resulted in a higher rate of food consumption and, in turn, led to a higher PCB accumulation rate. Females grew substantially faster than males, and therefore the PCBs were more “diluted” in the larger body masses of the females. Both freshwater and marine fish appear to share this characteristic of greater whole-fish ƩPCB in males compared with females, as this characteristic has now been documented for walleye, lake trout, coho salmon, burbot, sea lamprey, cisco, lake whitefish, and summer flounder. We recommend that a new line of research be initiated, as a follow up to our study, to more accurately account for the difference in energy expenditure rates between the sexes of summer flounder. To accomplish this goal, differences in both activities and SMRs between the sexes would need to be quantified.

## Supporting Information

S1 File Whole-fish ƩPCB for summer flounder from New Jersey coastal waters, November 2013. (XLSX)

S2 File Whole-fish PCB congener concentrations for summer flounder from New Jersey coastal waters, November 2013. (XLSX)

S3 File ƩPCB concentrations in somatic tissue and ovaries of female summer flounder from New Jersey coastal waters, November 2013. (XLSX)
